# Avoidant personality disorder through the lens of ICD 11

**DOI:** 10.1192/j.eurpsy.2024.1360

**Published:** 2024-08-27

**Authors:** A. Bogdanovska Toskic, S. Arsova Hadzi-Angjelkovska

**Affiliations:** ^1^Psychiatry, General Hospital Kumanovo, Kumanovo; ^2^Psychiatry, University Clinic of Psychiatry, Skopje, North Macedonia

## Abstract

**Introduction:**

With the new dimensional diagnosis of personality disorders in ICD 11, the categorical model has been abandoned. The types of personality disorders in the new dimensional model should show certain common characteristics. According to the recognition of the common characteristics of individual types of personality disorders, as well as determining the severity, a transition from the categorical to the dimensional diagnostic system can be made.

**Objectives:**

To analyze and present the trait domains specifiers in persons with avoidant personality disorder and to facilitate the adoption of the new diagnostic criteria.

**Methods:**

An unsystematized literature review was made, with key words: avoidant personality disorder, ICD 11, ICD 10, traits; and a case was presented.

**Results:**

This is the case of a 26-year-old student who has had no friends since his school days. During his secondary education, on the initiative of another person, he got together with several other people, but he was not fully accepted. During the studies, the communication with the colleagues took place only at the university and around the responsibilities. About a year ago, he had reduced willpower and suicidal thoughts, when he took antidepressant and adjuvant antipsychotic therapy for some time. He is now being examined due to severe tension, dissatisfaction, lack of friends, repeated suicidal ideation. According to researches, people with avoidant disorder have prominent trait domains – negative affectivity, detachment and reduced dissociality (Bach *et al*. BMC Psychiatry 2018; 18:351), negative affectivity, detachment and anankastia (Simon *et al*., Front. Psychiatry 2023, 14:1175425), negative affectivity and detachment (Bach *et al*. Borderline Personality Disorder and Emotion Dysregulation 2022, 9:12). In our case, assessments of trait domains were made with PSQ-11 and PiCD. On the PSQ-11, an increase in the negative affectivity, detachment and anankastia on critical score was obtained, while on the PiCD, an increase in negative affectivity, detachment, anankastia, and a decrease in dissociality was obtained. Mild personality disorder was scored on the Rating Scales for Severity of Disorder (SASPD, LPFS-BF 2.0).

**Conclusions:**

The types of personality disorder can be represented by certain common trait domains specifiers, which will be useful in adopting the diagnostic criteria in ICD 11 for personality disorder. Assessment of the severity of the disorder provides additional information on treatment strategies and prognosis. The most significant features of avoidant personality disorder are negative affectivity and detachment, while anankastia is on the borderline score and has a reduction in dissociality.

**Disclosure of Interest:**

None Declared

